# Work accidents registered in the Brazilian social security system between 2016 and 2020: a descriptive analysis

**DOI:** 10.47626/1679-4435-2023-1215

**Published:** 2024-11-14

**Authors:** Daniel Osti de Barros, Leopoldo Silva Oliveira, Josierton Cruz Bezerra, Eduardo Costa Sá

**Affiliations:** 1 Departamento de Medicina Legal, Bioética, Medicina do Trabalho e Medicina Física e Reabilitação, Faculdade de Medicina, Universidade de São Paulo, São Paulo, SP, Brasil; 2 Universidade Potiguar, Natal, RN, Brasil; 3 Departamento de Patologia, Escola Paulista de Medicina, Universidade Federal de São Paulo, São Paulo, SP, Brasil

**Keywords:** occupational accidents, work accidents in Brazil, occupational health, social security, prevalence, acidentes de trabalho, acidentes de trabalho no Brasil, saúde ocupacional, previdência social, prevalência

## Abstract

**Introduction:**

More than 500,000 work accidents were registered each year in Brazil from 2016 to 2018, representing more than BRL 300 million in expenditures.

**Objectives:**

To analyze the prevalence of work accidents in Brazil between 2016 and 2020 according to geographic region, age group, and sex and analyze the prevalence according to the cause and economic activity type.

**Methods:**

Descriptive, cross-sectional study based on data from the Social Security Statistics Yearbook.

**Results:**

The work accident rate between 2016 and 2019 was lower than in previous years. The Southern and Southeastern regions had the highest prevalence of work accidents (11.7/1,000 workers and 9.10/1,000 workers, respectively), while the Northeastern region had the lowest rate (6.22/1,000 workers). There was a greater reduction in work accidents among men than women. The most prevalent types of work accidents involved injuries, poisoning, and other external causes, in addition to diseases of the musculoskeletal system and connective tissue.

**Conclusions:**

Record keeping about work accidents must be improved, given that informal workers are not considered in social security data. More accurate data can also help increase prevention efforts, and can lead to more effective occupational health and safety policies to further reduce the work accident rate.

## Abstract (pt)

**Introdução:**

No Brasil, foram registrados mais de 500 mil acidentes de trabalho por ano no período entre 2016 e 2018, com custo anual superior a R$ 300 milhões.

**Objetivos:**

Analisar a prevalência dos acidentes de trabalho no Brasil entre os anos de 2016 e 2020 segundo as variáveis regiões geográficas, faixa etária e sexo e analisar a sua prevalência de acordo com as causas e o ramo de atividade econômica no mesmo período.

**Métodos:**

Tratou-se de um estudo descritivo, transversal, com análise de série temporal do Anuário Estatístico da Previdência Social no Brasil.

**Resultados:**

A partir da análise, foram identificadas menores taxas de acidentes de trabalho entre 2016 e 2019 em comparação com anos anteriores. As regiões Sul e Sudeste apresentaram-se como as localidades com maior prevalência dos acidentes de trabalho: 11,7/1.000 trabalhadores e 9,10/1.000 trabalhadores, respectivamente. Já o Nordeste demonstrou menor número de acidentes, com 6,22/1.000 trabalhadores. Em relação ao sexo, houve redução na prevalência dos acidentes de trabalho, sendo as maiores diminuições para o gênero masculino. As maiores prevalências de acidentes de trabalho por causa relacionam-se, respectivamente, a lesões, envenenamentos e outras consequências de causas externas e doenças do sistema osteomuscular e do tecido conjuntivo.

**Conclusões:**

É necessário melhorar os registros de acidentes de trabalho devido à subnotificação dos trabalhadores informais não considerados nos anuários. Além disso, dados mais apurados possibilitam a ampliação de políticas de prevenção, saúde e segurança no trabalho, para, então, reduzir os números desses acidentes.

## INTRODUCTION

To characterize work accidents (WA), it is necessary to understand the profile of the involved population, considering their location, race, age, education level, and other variables. The causes and consequences of WA must also be investigated. Brazilian data collected between 2008 and 2014 indicated a reduction in WA compared to previous years,^1^ with the highest prevalence occurring in the Southeastern and Southern regions of the country among men and among workers aged 20-49 years.^2^ According to data from the Brazilian social security system, more than 600,000 WA were registered each year between 2016 and 2018, at an annual cost > BRL 300 million.^3^ The population most affected by WA is Black men between 18 and 39 years of age. People with a higher education level had a lower frequency of WA. Therefore, the sample profile for WA is characterized by workers with a lower education who reside in Southeastern Brazil.^4^

Between 2008 and 2014, injuries, poisonings, and other external causes were the main causes of WA registered in the Brazilian social security system (70.8% of cases), followed by work-related musculoskeletal disorders (16%). The processing industry and commerce, including trade and repair of motor vehicles and motorcycles, were the two main economic activities in which WA occurred, representing 31.7 and 13.1% of cases, respectively.^2^

According to the tenth revision of the International Statistical Classification of Diseases and Related Health Problems (ICD-10), determining the association between disease groups and economic activities is essential for a better understanding of WA. Studies have also shown the importance of understanding the relationship between WA and the political economy for a better understanding of worker health.^5^

Although these numbers are important for a better understanding of the national WA scenario, data from the Brazilian Institute of Geography and Statistics’ National Health Survey must also be considered. Compared to official records from the former Ministry of Social Security, 7 times more WA have been registered in the National Social Security Institute database. This difference suggests that the number of cases is underreported, as well as the existence of a significant number of informal workers.^6^ Thus, it is important to understand the prevalence of WA registered in the Brazilian social security system between 2016 and 2020 according to cause, economic activity, and sociodemographic data. Understanding the prevalence of WA can stimulate public and private sector policies, enabling a better allocation of financial and human resources to prevent WA.

The objective of this study was to analyze the prevalence of WA registered in the Brazilian social security system between 2016 and 2020 according to geographic region, age group, and sex, and to determine the prevalence according to cause and type of economic activity.

## METHODS

### ETHICAL ASPECTS

According to National Health Council Resolution 510/2016, since this study is based on information from publicly accessible databases, which, in themselves, do not allow personal identification, under the terms of Law 12,527, the present study was not submitted to an institutional research ethics committee or the National Research Ethics Commission.

### STUDY TYPE, LOCATION, PERIOD, AND SAMPLE

This cross-sectional, descriptive study investigated the *Anuário Estatístico da Previdência Social* (Social Security Statistical Yearbook) from 2016 to 2020, which included the initial implementation of a new WA reporting methodology called the *Nexo Técnico Epidemiológico Previdenciário* (Social Security Epidemiological Technical Nexus), which began in April 2006.

### STUDY PROTOCOL

Data were collected from the social security platform and the 2016-2020 statistics yearbooks^3^ on March 7, 2022. The collected data were copied and transferred to Excel spreadsheets, in which the prevalence of WA was calculated as the number of new cases registered, divided by the number of workers registered in the system in that location and year, multiplied by 1,000 workers. The data for the numerator were collected from the social security website, while the denominator was derived from annual consolidated Ministry of Labor and Social Security data from 2016 to 2020.^3^

Prevalences were calculated for the entire country and according to region, sex, and age group (≤ 19 years, 20-49 years, 50-64 years, and ≥ 65 years). Data ignored in each of these categories were not considered in the present study. The prevalence of WA was calculated between 2016 and 2020 according to ICD-10 disease group designation. The causes of WA and the major types of economic activity described in the *Classificação Nacional de Atividades Econômicas* (National Classification of Economic Activities) 2.0 were selected due to the impossibility of determining the function of the affected worker, since such data is not provided by the social security system. The top 5 economic activities and the ICD-10 designations with the highest WA prevalence were used in the study. Prevalence was calculated by dividing the number of registered WA in each ICD-10 disease group and each economic activity subgroup. This number was then divided by the total number of WA registered in the social security system from 2016 to 2020 and multiplied by 100.

## RESULTS

**Figure 1:**
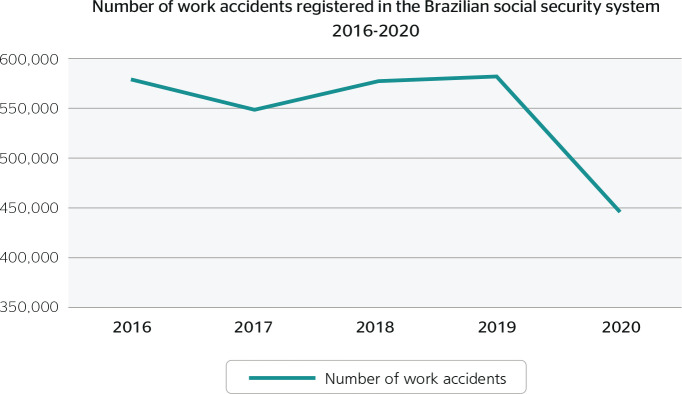
Distribution of workers and work accidents registered in the Brazilian social security system from 2016 to 2020.

**Figure 2:**
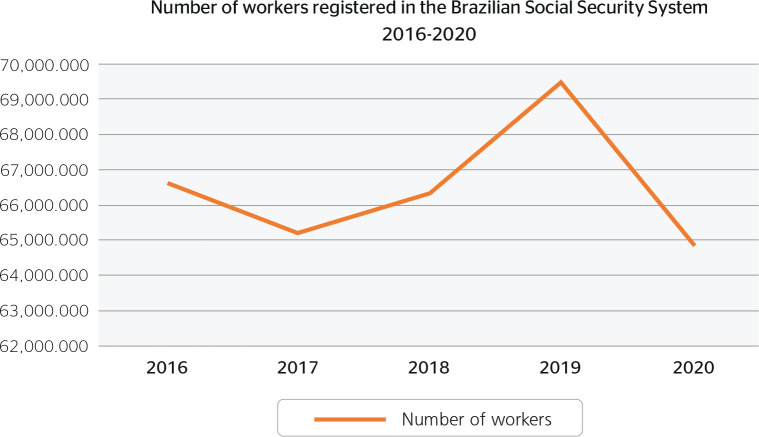
Distribution of the total number of workers registered in the Brazilian social security system from 2016 to 2020.

Each year from 2016 to 2019, more than 500,000 WA were registered in the Brazilian social security system. The number decreased significantly in 2020 to 445,814 (Figure 1 and Table 1). Nevertheless, there was also an overall downward trend in prevalence between 2016 and 2020, with a 21.85% reduction: from 0.88/1,000 workers in 2016 to 0.69/1,000 workers in 2020 (Table 2). The total number of workers registered annually in the social security system fluctuated between 67 and 65 million, reaching a maximum of 69.4 million in 2019 and a minimum of 64.9 million in 2020 - a decrease of 4.5 million workers (Figure 2 and Table 1).

**Table 1:** Distribution of work accidents and workers registered in the Brazilian social security system according to sex, geographic region, and age group, 2016-2020

Ecological unit of analysis	2016	2017	2018	2019	2020
Brazil					
Accidents	585,626	557,626	576,951	586,857	445,814
Workers	66,652,055	65,232,942	66,339,030	69,481,633	64,924,484
Sex					
Male					
Accidents	389,111	369,701	380,559	386,601	200,111
Workers	36,016,133	35,198,302	35,751,348	37,412,300	35,182,047
Female					
Accidents	196,493	187,914	196,370	198,804	149,595
Workers	30,540,444	29,946,829	30,422,800	31,855,717	29,538,703
Region					
South					
Accidents	131,193	126,179	132,481	135,672	102,669
Workers	11,849,611	11,743,533	12,036,461	12,642,713	11,896,386
Southeast					
Accidents	314,129	296,406	306,508	314,550	237,653
Workers	34,534,688	33,845,212	34,401,717	35,825,539	33,387,164
Midwest					
Accidents	44,523	44,387	46,654	47,608	38,263
Workers	5,653,660	5,447,337	5,569,006	5,880,766	5,449,614
Northeast					
Accidents	70,306	66,082	65,880	65,011	47,970
Workers	11,299,512	10,980,768	11,100,369	11,590,950	10,721,059
North					
Accidents	25,475	24,572	25,428	24,016	19,286
Workers	3,166,123	3,104,318	3,115,273	3,274,139	3,117,355
Age range (years)					
≤ 19					
Male					
Accidents	10,076	8,546	9,373	8,697	6,770
Workers	1,232,356	1,082,701	1,062,823	1,050,266	920,196
Female					
Accidents	3,450	3,060	3,123	2,938	1,941
Workers	995,008	881,549	871,061	863,486	743,506
20-49					
Male					
Accidents	319,516	303,872	313,668	318,631	245,591
Workers	27,761,790	26,950,026	27,332,100	28,594,804	26,763,325
Female					
Accidents	162,041	153,594	161,036	163,822	125,435
Workers	23,528,589	22,969,498	23,258,483	24,339,734	22,404,253
50-64					
Male					
Accidents	56,859	54,684	54,783	56,373	40,767
Workers	6,291,928	6,397,425	6,536,659	6,899,157	6,633,253
Female					
Accidents	30,069	30,238	30,997	32,063	21,579
Workers	5,528,645	5,623,527	5,775,016	6,091,044	5,817,998
≥ 65					
Male					
Accidents	2,659	2,591	2,733	2,894	1,782
Workers	729,933	767,976	818,437	867,962	865,151
Female					
Accidents	931	1,021	1,212	1,286	639
Workers	434,129	471,649	517,307	561,569	572,905

Source: Brazilian Social Security Statistics Yearbook.

**Table 2:** Distribution of work accidents (per 1,000 workers) registered in the Brazilian social security system according to geographic region, sex, and age group from 2016 to 2020

Ecological unit of analysis	2016	2017	2018	2019	2020	% reduction
Brazil	0.88	0.85	0.87	0.84	0.69	21.85
Region						
South	11.07	10.74	11.01	10.73	8.63	22.05
Southeast	9.10	8.76	8.91	8.78	7.12	21.75
Midwest	7.88	8.15	8.38	8.10	7.02	10.84
Northeast	6.22	6.02	5.93	5.61	4.47	28.09
North	8.05	7.92	8.16	7.34	6.19	23.11
Sex						
Male	10.80	10.50	10.64	10.33	5.69	47.35
Female	6.43	6.27	6.45	6.24	5.06	21.29
Age range (years)						
≤ 19						
Male	8.18	7.89	8.82	8.28	7.36	10.02
Female	3.47	3.47	3.59	3.40	2.61	24.71
Total	6.72	5.91	6.46	6.08	5.24	22.02
20-49						
Male	11.51	11.28	11.48	11.14	9.18	20.27
Female	6.89	6.69	6.92	6.73	5.60	18.71
Total	9.39	9.16	9.38	9.11	7.55	19.60
50-64						
Male	9.04	8.55	8.38	8.17	6.15	31.99
Female	5.44	5.38	5.37	5.26	3.71	31.80
Total	7.35	7.06	6.97	6.81	5.01	31.84
≥ 65						
Male	3.64	3.37	3.34	3.33	2.06	43.46
Female	2.14	2.16	2.34	2.29	1.12	47.99
Total	3.08	2.91	2.95	2.92	1.68	45.45

Source: Brazilian Social Security Statistics Yearbook.

The Southeast, the country’s main economic region, had the second highest prevalence of WA, surpassed only by the Southern region. In 2016, the prevalence was 9.10/1,000 workers and 11.07/1,000 workers in the Southeastern and Southern regions, respectively. There was a downward trend in both regions, reaching 7.12/1,000 workers in the Southeastern region and 8.63/1,000 workers in the Southern region in 2020: reductions of 21.75 and 22.05%, respectively. The Northeastern region had the greatest reduction in WA prevalence, followed by the Northern region. In 2016, the prevalence was 6.22/1,000 workers in the Northeastern region and 8.05/1,000 workers in the Northern region. In 2020, these numbers fell to 4.47/1,000 workers in the Northeastern region and 6.19/1,000 workers in the Northern region, resulting in reductions of 28.09% and 23.11% in these regions, respectively, during this period.

The Midwestern region had the smallest reduction in WA between 2016 and 2020, decreasing from 7.88/1,000 workers to 7.02/1,000 workers (-10.84%)(Tables 1 and 2).

The prevalence of WA decreased in both sexes, although the reduction was more significant among men, reducing from 10.8/1,000 workers to 5.69/1,000 workers (47.35%). Among women, the prevalence decreased from 6.43/1,000 workers to 5.06/1,000 workers (-21.29%).

Regarding age groups, the greatest reduction in WA prevalence occurred among workers aged 50-64 years and those aged ≥ 65 years, with similar reductions in both sexes. In the 50-64 age group, there was a reduction of 31.99% for men and 31.8% for women, while in the ≥ 65 group there was a reduction of 43.46% for men and 47.99% for women. In the 20-49 age group, both sexes had a similar reduction, from 11.51/1,000 workers to 9.18/1,000 workers among men (-20.27%) and 6.98/1,000 workers to 5.60/1,000 workers among women (-18.7%).

In the ≤ 19 age group, the prevalence among women decreased from 3.47/1,000 workers to 2.61/1,000 workers (-24.71%), while among men it decreased from 8.18/1,000 workers to 7.36/1,000 workers (-10.02%) (Table 2).

**Figure 3:**
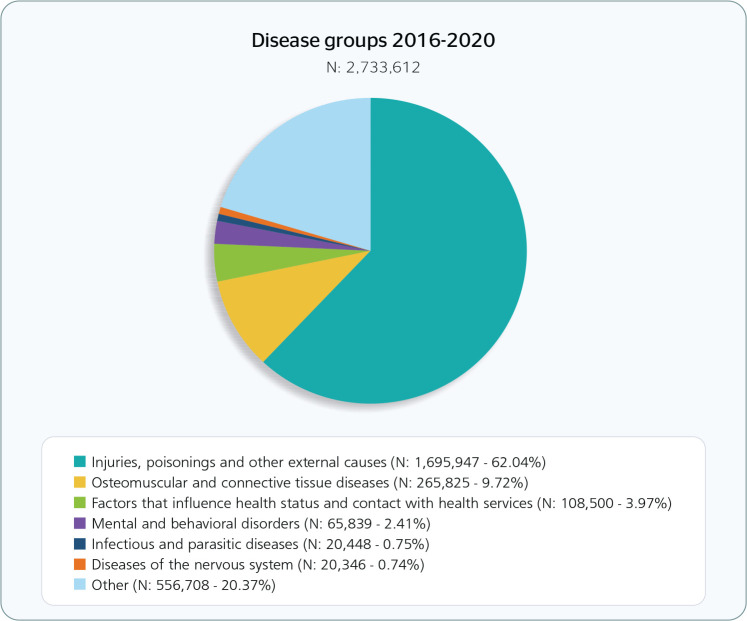
Main causes of work accidents registered in the Brazilian social security system according to the tenth revision of the International Statistical Classification of Diseases and Related Health Problems, 2016-2020.

The five main causes of WA in absolute numbers were injuries, fractures, and trauma to the wrist and hands, back pain, dislocations, sprains, and strains in the joints and ligaments of the foot and ankle (Figure 3 and Table 3).

**Table 3:** Main causes of work accidents registered in the Brazilian social security system, according to the tenth revision of the International Statistical Classification of Diseases and Related Health Problems, 2016 to 2020

Disease group	n	%	Cause of the accident	n	% in this group of causes
Injuries, poisonings, and other external causes	1,695,947	62.04	Wrist and hand injuries	266,400	15.71
			Fractures of the wrist and hand	167,579	9.88
			Dislocations, sprains, and strains of the joints and ligaments of the foot and ankle	128,314	7.57
			Superficial trauma to the wrist and hand	120,082	7.08
			Leg (including ankle) fractures	91,449	5.39
Osteomuscular and connective tissue diseases	265,824	9.72	Back pain	98,149	36.92
			Shoulder injuries	60,965	22.93
			Other joint disorders not classified elsewhere	32,749	12.32
			Synovitis and tenosynovitis	27,187	10.23
			Other soft tissue disorders not classified elsewhere	15,451	5.81
Factors that influence health status and contact with health services	108,500	3.97	Contact with and exposure to communicable diseases	59,991	55.29
			Examination and observation for other reasons	13,621	12.55
			General examination and investigation of people without complaints or reported diagnosis	11,952	11.02
			Occupational exposure to risk factors	2,817	2.60
Mental and behavioral disorders	65,839	2.41	Reactions to severe stress and adjustment disorders	39,474	59.96
			Other anxiety disorders	15,622	23.73
			Depressive episodes	10,743	16.32
Infectious and parasitic diseases	20,448	0.75	Unspecified viral diseases	20,448	100.00
Diseases of the nervous system	20,346	0.74	Mononeuropathies of the upper limbs	20,346	100.00

Source: Brazilian Social Security Statistics Yearbook.

According to social security system records, around 62% of WA were classified as injuries, poisonings, and other external causes. Injuries and fractures to the hands and wrists were prominent in this group (Table 3). Approximately 10% of WA were associated with diseases of the musculoskeletal system and connective tissue, especially back pain (Table 3). At a lower prevalence, around 4% of WA were due to factors that influence health status and contact with health services, especially exposure to communicable diseases. Mental and behavioral disorders comprised 2.41% of WA cases, mainly severe reactions to stress and adjustment disorders (Table 3), while 0.75% were due to infectious and parasitic diseases, with unspecified viral diseases being the most common type.

**Figure 4:**
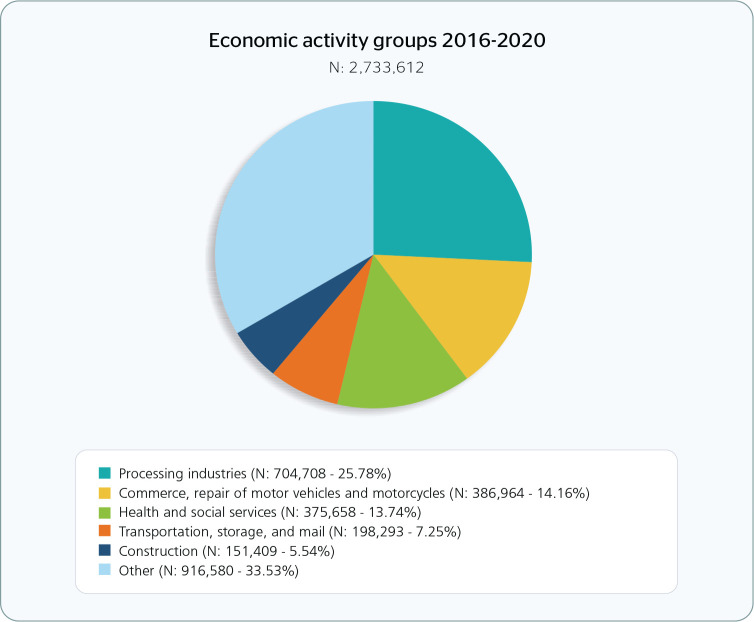
Distribution of work accidents registered in the Brazilian social security system according to the main economic activities listed in the National Classification of Economic Activities 2.0, 2016-2020.

Approximately 26% of WA occurred in processing industries (Figure 4 and Table 4), among which the slaughter of pigs, poultry, and other small animals stood out (Table 4). A total of 4.16% of WA cases occurred in the commerce (including the repair of motor vehicles and motorcycles) sector, with the retail trade of general merchandise, hypermarkets and supermarkets being the most affected segment.

**Table 4:** Distribution of work accidents registered in the Brazilian social security system according to the main categories in the National Classification of Economic Activities 2.0, 2016-2020

Activity	n	%	Most frequent occupations	n	% in this activity
Processing industries	704,708	25.78	Slaughter of pigs, poultry, and other small animals	56,963	8.08
			Slaughter of livestock, except pigs	35,244	5.00
			Raw sugar manufacture	29,085	4.13
			Manufacture of plastic artifacts not previously specified	17,768	2.52
			Manufacture of furniture, predominantly wood	16,313	2.31
Commerce, repair of motor vehicles and motorcycles	386,964	14.16	Retail trade of general merchandise, food products - hypermarkets and supermarkets	108,169	27.95
			Retail trade of hardware, wood, and construction materials	25,856	6.68
			Sale of motor vehicle parts and accessories	18,739	4.84
			Retail trade of pharmaceutical products for human and veterinary use	12,599	3.26
			Wholesale beverage trade	12,508	3.23
Health and social services	375,658	13.74	Hospital care activities	283,266	75.41
			Outpatient care activities performed by doctors and dentists	23,664	6.30
			Diagnostic and complementary therapy activities	22,840	6.08
			Health care activities not previously specified	13,368	3.56
			Health management support activities	13,053	3.47
Transportation, storage, and mail	198,293	7.25	Highway freight transportation	63,676	32.11
			Postal activities	50,763	25.60
			Public municipal transportation of passengers with a fixed itinerary	25,524	12.87
			Storage	7,679	3.87
			Metro rail passenger transport	6,372	3.21
Construction	151,409	554	Building construction	46,554	30.75
			Construction for the generation and distribution of electrical energy and telecommunications	19,554	12.91
			Incorporation of real estate projects	15,477	10.22
			Construction of highways and railways	13,552	8.95
			Electrical installations	9,317	6.15

Source: Brazilian Social Security Statistics Yearbook.

The prevalence of WA in the field of health and social services was around 14%, with hospital care being the most common source of WA (Table 4). It is worth noting that from 2019 to 2020 the only increase in WA occurred in the health and social services sector.

A total of 7.25% of the WA occurred in the transportation, storage, and postal activities sector, especially highway freight transportation (Table 4); while 5.54% occurred in the construction sector, especially building construction.

## DISCUSSION

The data indicated a general downward trend in WA registered in the Brazilian social security system (Figure 1). This trend was observed in all regions of the country, including both sexes and all age groups. These results are in line with similar findings from previous studies indicating a reduction in WA in Brazil during specific periods, such as from 1998 to 2008,^7^ from 2008 to 2014,^2^ and from 2008 to 2013.^1^

However, in 2019 the number of workers increased, although this increase was not accompanied by a proportional increase in WA (Table 1 and Figures 1 and 2). One explanation for this could be the increase in the tertiary sector, especially commerce, which entails a low risk of WA. The impact of the 2019 pension reform may also have had some effect, although further studies are needed for greater understanding of this event. Although the total number of WA is still high, corporate preventive measures and public policies aimed at worker health and safety have proven effective in reducing the total number of WA. However, it is important to highlight the persistence of underreporting, whether due to omission by responsible parties or to limited reporting about informal workers.^6^

According to our results, the main economic regions of the country also had the highest prevalence of WA: the Southeastern region had the highest absolute number of cases, while the Southern region had the highest rate. This is in line with other studies that identified a higher prevalence of deaths due to WA in the state of Minas Gerais between 1996 and 2006, which was above the national average.^8^ Similar values and trends were found by Bezerra et al.,^2^ who attributed this situation not only to the greater economic development of these regions, resulting in a greater risk of WA, but also to greater WA notification in these regions, leading to a higher reported prevalence than in other regions. Economically developed regions also have more processing industries, more commerce (including repair of motor vehicles and motorcycles), and more hospital care activities, which, together, account for > 50% of WA cases (Table 3).

We also analyzed the difference in WA between men and women, finding that, despite a reduction in both sexes, men were more affected than women between 2016 to 2019, which is in line other studies.^2,9,10^ This trend has been observed in other countries with similar cultures and economies to Brazil, such as Ecuador and Colombia, where the prevalence of WA was higher among men, although significant sex differences were not found in Chile.^11^ However, in Brazil in 2020, the WA prevalence between the sexes was quite close. This might be explained by the health measures adopted to combat the COVID-19 pandemic, which restructured the national work environment, keeping only essential economic activities in operation, especially health-related activities.

This more equitable prevalence may be attributable to the greater number of health workers, especially the hospital sector.^12^ This explanation is supported by other studies, such as one conducted at a teaching hospital in Curitiba, in Southern Brazil, which found that more than 80% of nurses were women.^13^ Furthermore, a review found an increase in COVID-19 infection among female workers, a phenomenon observed in 70% of the reviewed articles, especially those from China, the United States, and Italy.^14^

Regarding WA and age groups, there was a higher prevalence among workers aged 20 to 49 years, both men and women. This is due to the fact that this age group represents the bulk of the economically active population, totaling more than 49 million workers (Table 1) who are, consequently, more exposed to occupational risk.^2^ These results are in agreement with other studies.^8,15^

The highest prevalence of WA in our results was associated with injuries, poisonings, and other external causes. This covers a variety of injuries such as bruises, fractures, dislocations, and trauma. This is explained by the fact that these injuries are most often linked to the work environment, and can occur while working or when commuting to and from work. These circumstances are less prone to underreporting, as they are directly related to work activity and are more likely to result in serious injuries or fatal accidents, physically disabling workers and interrupting the production process.

Diseases of the musculoskeletal system and connective tissue were the second most frequent type. This category is directly related to work environment conditions, including infrastructure and ergonomic factors. The connection between these diseases and the work environment raises the issue of ergonomics as a potential cause of work-related health problems, although this issue is sometimes neglected.^2^ Among diseases of the musculoskeletal system and connective tissue, back pain stood out as the main cause of WA, representing approximately 37% of cases within this group.

The third most frequent disease group was factors that influence health status and contact with health services, representing 3.97% of WA between 2016 and 2020, an increase over 2008-2014 (2.19%).^2^ It is important to highlight that the main cause in this group is contact with and exposure to communicable diseases, contributing to 55.29% of WA. There is growing concern about reporting these types of WA due to the risk they pose to workers, especially workers frequently exposed to biological materials.^2,16^ A related group, which ranked fifth among the main causes of WA, was infectious and parasitic diseases. In this group, the only recorded cause is unspecified viral diseases, corresponding to 100% of the WA in this group. It is important to note that all records of this cause occurred in the same year that COVID-19 infection began, which reinforces the importance of reporting infectious diseases in the workplace.

The fourth most frequent disease group was mental and behavioral disorders, which, although representing a small percentage in relation to the other groups, is important due to its connection with contemporary work organization.^17^ The work process, often marked by a demand for increasing results, leads to a series of challenges, including work overload, social breakdown, precarious labor relations, increased risk of wage losses, and even symbolic violence, contributing to a relationship between work and depression.^2,17,18^ Thus, in this group, reactions to severe stress and adjustment disorders are the most frequent causes of WA, followed by other anxiety disorders and depressive episodes. To minimize these effects on worker quality of life, mental health and worker health policies must be discussed and implemented, increasing prevention measures and surveillance, while reducing the harmful effects of work conditions.

Regarding the economic activities analyzed in this study, the high prevalence of WA in the processing industry sector stands out. It is notable that these industries, which are mainly concentrated in the Southern and Southeastern regions of Brazil, also had the highest number of WA in this study. A previous study that analyzed Social Security Statistics Yearbooks data from the end of the first decade and the beginning of the second decade of the 21st century^2^ also found that processing industries had the highest prevalence of WA in Brazil. In addition, an Ecuadorian study found that processing industries had the highest prevalence of WA, approximately 26% of all WA.^19^

Within this sector, the frequency of WA in occupations related to the slaughter of pigs, poultry and other small animals should be highlighted. The high rate of accidents in these occupations highlights the precarious working conditions to which slaughterhouse employees are exposed. This includes intense work pace, the lack of ergonomic equipment design, and the high employee turnover rate.^20^

It is interesting to note that raw sugar manufacturing, which in previous years had the highest rate of WA in the processing industry,^2^ experienced a significant reduction (> 30%) in WA in relation to total cases during the study period. This reduction could be attributed to the mechanization of sugarcane harvesting, which has intensified in recent years. This process has reduced the number of workers involved in this occupation.^21^ Thus, it has reduced the physical, chemical, and mechanical risks for workers, although it has not completely eliminated the biological and chemical risks. In addition, new risks have emerged, such as noise and vibrations, which have impacted worker health.^22^ It is worth noting that the mechanization process in the sugar and alcohol sector has reduced WA by both reducing the risks associated with manual harvesting and by reducing the number of workers employed in the sector.

Regarding commerce, including the repair of motor vehicles and motorcycles, it is notable that this sector had the second highest prevalence of WA in this study. It is interesting that, although the total number of WA in this field reduced during the study period, it remained stable between 2008 and 2014.^2^ The WA rate in the transportation, storage, and postal activities sector was also stable between 2008 and 2014.^2^

The third highest prevalence of WA in this study was health and social services, especially hospital care activities. It is important to highlight the importance of activities related to human health, due to the possibility of WA related to exposure to biological agents.^23^ According to the literature, the majority of these accidents are related to the handling of sharp materials, such as needles and ampoules, which represent a great risk for health care professionals, especially those involved in direct patient care.^24,25^

Another factor that could contribute to a higher prevalence of WA in this field of activity is related to the emergence of COVID-19 infection in 2020, both globally and in Brazil. The fight against COVID-19 resulted in work overload and required both psychological and physical skills from health care professionals.^26,27^ It is important to note that the only increase in WA during the study period occurred in the health and social services sector between 2019 and 2020. This percentage increased from 8.71% between 2008 and 2014^2^ to 13.74% between 2016 and 2020, which further highlights the importance of analyzing this sector of activity.

Activities related to the construction sector, especially building construction, had the fifth highest prevalence of WA in this study. The reduction in WA in this field of activity (5.4% between 2016 and 2020 vs. 7.93% between 2008 and 2014)^2^ might be attributable to the crisis in the Brazilian real estate sector between 2015 and 2017, leading to fewer active companies and professionals in construction.^28^ Furthermore, the large number of work absences in this sector during the COVID-19 pandemic in 2020^29^ may also have contributed to this decrease. Studies in other countries have found higher rates of WA than ours, for example, Colombia (28%), Ecuador (17.47%), Argentina (15%) and Spain (14%) between 2016 and 2021^19^, as well as between 2013 and 2017.^30^

### STUDY LIMITATIONS

Since the present study only examined data from the Brazilian social security system, it does not consider unregistered (informal) workers. The possibility of underreporting data must be considered, even in official records. However, the social security database is an important source of data widely used in various scientific studies.

## CONCLUSIONS

Although Brazil had a high prevalence of WA between 2016 and 2020, there was a trend towards reduction in overall numbers, which was more pronounced in the Southern and Southeastern regions. A downward trend was also observed among men, among workers aged 20 to 49 years, in the processing industry, and in health and social services sectors, especially occupations related to hospital care, which presented the highest absolute number of WA during the study period. It is important to note that notifications increased in this sector in 2020, which may have been related to the COVID-19 pandemic.

The COVID-19 pandemic also had a significant impact on WA related to infectious and parasitic diseases, making this disease group one of the most common types of WA in this study. The increased reporting of this type reflects greater awareness and concern about workplace safety in relation to infectious diseases in recent years.

Overall, it is crucial to improve both the quantity and quality of WA records to avoid underreporting. This will lead to a more accurate database and, consequently, allow the development of more effective prevention policies and occupational health and safety policies to reduce the number of WA.
